# Clinical spectrum and prevalence associations of chorioretinal damage in high myopia: a retrospective cross-sectional analysis

**DOI:** 10.3389/fmed.2026.1752116

**Published:** 2026-02-17

**Authors:** Guowei Li, Ye Wang, Xueyi Chen

**Affiliations:** 1 Lanzhou Aier Eye Hospital, Lanzhou, Gansu, China; 2 Gansu Aier Optometry Hospital, Lanzhou, Gansu, China

**Keywords:** chorioretinal damage, clinical outcomes, epidemiology analysis, myopia, predictors

## Abstract

**Background:**

High myopia is a growing public-health challenge linked to irreversible chorioretinal damage and vision loss. Understanding its clinical spectrum and identifying predictors of early structural injury are essential for risk-stratified surveillance. Despite progress in imaging and diagnostic classification, population-level evidence describing lesion patterns and predictive factors in high-burden clinical settings remains limited.

**Methods:**

The cross-sectional retrospective study evaluated 1,420 patients (2,610 eyes) with high myopia examined between 2015 and 2024 at Lanzhou Aier Eye Hospital. Eligibility was based on refractive or biometric criteria and availability of multimodal imaging. Chorioretinal changes were graded using the ATN classification. Descriptive statistics summarized demographics and imaging findings. Prevalence of each lesion type was estimated with confidence intervals. Univariable comparisons and multivariable logistic regression with clustered variance identified independent predictors. Model calibration and discrimination were assessed, with prespecified sensitivity analyses based on axial length, age strata, and exclusion of treated choroidal neovascularization. Missing data were imputed using chained equations.

**Results:**

Chorioretinal damage was documented in 40.0% of eyes. Diffuse atrophy (16.1%), foveoschisis (11.9%), and patchy atrophy (8.2%) were the most frequent lesions. Older age, greater axial length, more negative spherical equivalent, and thinner choroid were independently associated with the presence of pathology. The final model demonstrated stable calibration and high discrimination across sensitivity analyses. Projected estimates indicated considerable population-level burden, particularly in individuals with axial length ≥32 mm.

**Conclusion:**

High-myopia eyes show substantial structural vulnerability, and key biometric parameters can support targeted early surveillance. The findings contribute new epidemiological evidence from a large clinical cohort and highlight practical risk-stratification markers suitable for public-health planning in regions facing rapid growth in myopia prevalence.

## 1 Introduction

High myopia has emerged as a growing public-health concern because of its rising prevalence and the disproportionate burden of vision-threatening complications that follow excessive axial elongation. Population-level estimates and trend analyses demonstrate a steep recent increase in myopia and a substantive rise in high myopia among children and young adults, with projections indicating continued growth in affected cohorts worldwide. The epidemiological expansion of high myopia translates to larger absolute numbers of eyes at risk for chorioretinal degeneration, with attendant increases in disability, healthcare utilization, and socioeconomic impact (1, 2).

The clinical spectrum of chorioretinal damage in highly myopic eyes encompasses a range of atrophic, tractional, and neovascular phenotypes that often coexist and drive progressive visual loss (3). Atrophic changes include tessellated fundus, diffuse and patchy chorioretinal atrophy, and macular atrophy; tractional entities include myopic traction maculopathy with foveoschisis and macular detachment; neovascular complications include myopic choroidal neovascularization that may produce fibrovascular scarring. Contemporary classification frameworks, such as the atrophy-traction-neovascularization (ATN) system and related staging schemes, permit standardized description of disease subtypes and severity and facilitate longitudinal assessment of progression and outcomes. Comprehensive clinical description must integrate multimodal imaging findings from fundus photography, optical coherence tomography, and angiography to capture the full phenotypic complexity (4, 5).

Risk predictors for chorioretinal damage in high myopia include biometric, demographic, and structural imaging markers that modulate susceptibility and temporal course. Longer axial length and greater myopic refractive error are consistently associated with higher risk and more severe maculopathy, and axial elongation rate predicts earlier onset and faster progression in younger cohorts. Age and baseline severity of myopic maculopathy also stratify progression risk. Emerging quantitative markers from OCT and vascular imaging, such as choroidal thinning, reduced choriocapillaris density, and alterations in deep capillary plexus metrics, show promise as mechanistic biomarkers that correlate with atrophy and tractional changes. Despite these insights, many studies remain cross-sectional, focused on selected clinical samples, or limited to single imaging modalities, constraining integrated risk modeling across populations (6, 7).

Recent work in systemic biomarker discovery illustrates how routine inflammatory indicators can stratify disease susceptibility, supporting the rationale for identifying objective predictors in high-risk ocular conditions (8, 9). Advances in ocular immunology and imaging, including detailed characterization of immune-mediated uveitis and the development of high-resolution microvascular and wavefront assessment tools, highlight how modern analytical approaches can refine detection of early structural changes in eyes vulnerable to chorioretinal injury (10–13).

The main objective of the research was to describe the clinical spectrum and rates of ATN-detected chorioretinal losses in a high myopic cohort of a tertiary eye care unit, and to estimate its connections with clinical and imaging variables routinely assessed. Instead of attempting to identify completely new biomarkers, this study itself was meant to reaffirm and generalize existing findings about high myopia, axial elongation, and chorioretinal pathology in an unrepresentatively representative group, and to translate it into a risk-stratification model that can be understood clinically in the form of standardized ATN grading and multimodal imaging. This work contributes novelty by combining: (1) a large retrospective sample spanning pediatric and adult age ranges to capture age-dependent patterns; (2) systematic application of an established classification to enable phenotype-specific risk modeling; and (3) integration of axial length, refractive error, OCT-derived structural metrics, and clinical variables into multivariable predictive models. The manuscript addresses a gap in the literature where prior reports have been limited by small cohorts, restricted age bands, or single-modality assessments, and where population-level retrospective analyses that relate standardized phenotypes to measurable predictors are scarce. The primary objective is to quantify the association strength of candidate predictors with discrete chorioretinal outcomes and to produce clinically applicable predictors that inform screening and follow-up strategies for eyes at highest risk of irreversible visual loss.

## 2 Methods

### 2.1 Study design and setting

This retrospective cross-sectional study was conducted at Lanzhou Aier Eye Hospital, a tertiary eye care center, which has an established electronic medical record (EMR) system. All clinical encounters, imaging records, and diagnostic measurements of patients attending the myopia and retina services between January 2015 and December 2024 were screened. For eligible patients, only baseline (first-visit) clinical and imaging data were extracted and analyzed to estimate the prevalence and clinical associations of chorioretinal damage; no longitudinal follow-up data or disease progression were evaluated. The study protocol was approved by the institutional ethics committee and adhered to the tenets of the Declaration of Helsinki. Only anonymized data were used to protect patient confidentiality.

### 2.2 Eligibility criteria

#### 2.2.1 Inclusion criteria

Patients that were screened were all patients that showed up in the tertiary ophthalmic center between January 2015 and December 2024. In the electronic medical archive, 3,200 patients whose diagnosis was recorded as myopia were found. Out of this sample, 1,870 patients (3,450 eyes) were found to have high myopia according to the preliminary definition of high myopia using refractive or biometric recordings. Spherical equivalent (SE) 0 or less and/or axial length (AL) 26.5 mm or more was considered high myopia as the International Myopia Institute (IMI) defined high myopia and pathologic myopia. The inclusion criteria were as follows: (1) refractive error spherical equivalent ≤ -6.00 diopters or (2) axial length 26.5 mm and (3) availability of good quality multimodal imaging session comprising of color fundus photography and OCT. SE and AL gave an opportunity to capture the structural and refractive components of high myopia, as well as in eyes with discrepant SE-AL profiles. To allow a valid evaluation of the progression or stability of chorioretinal changes, the minimum follow-up period of 6 months was also required, but in the current study, only baseline (first-visit) clinical and imaging data were obtained and evaluated within a retrospective cross-sectional study. Supplementary Table S1 represents the step by step selection of the eyes and patients out of initial screening to the final selection of the study.

#### 2.2.2 Exclusion criteria

**Figure 1:**
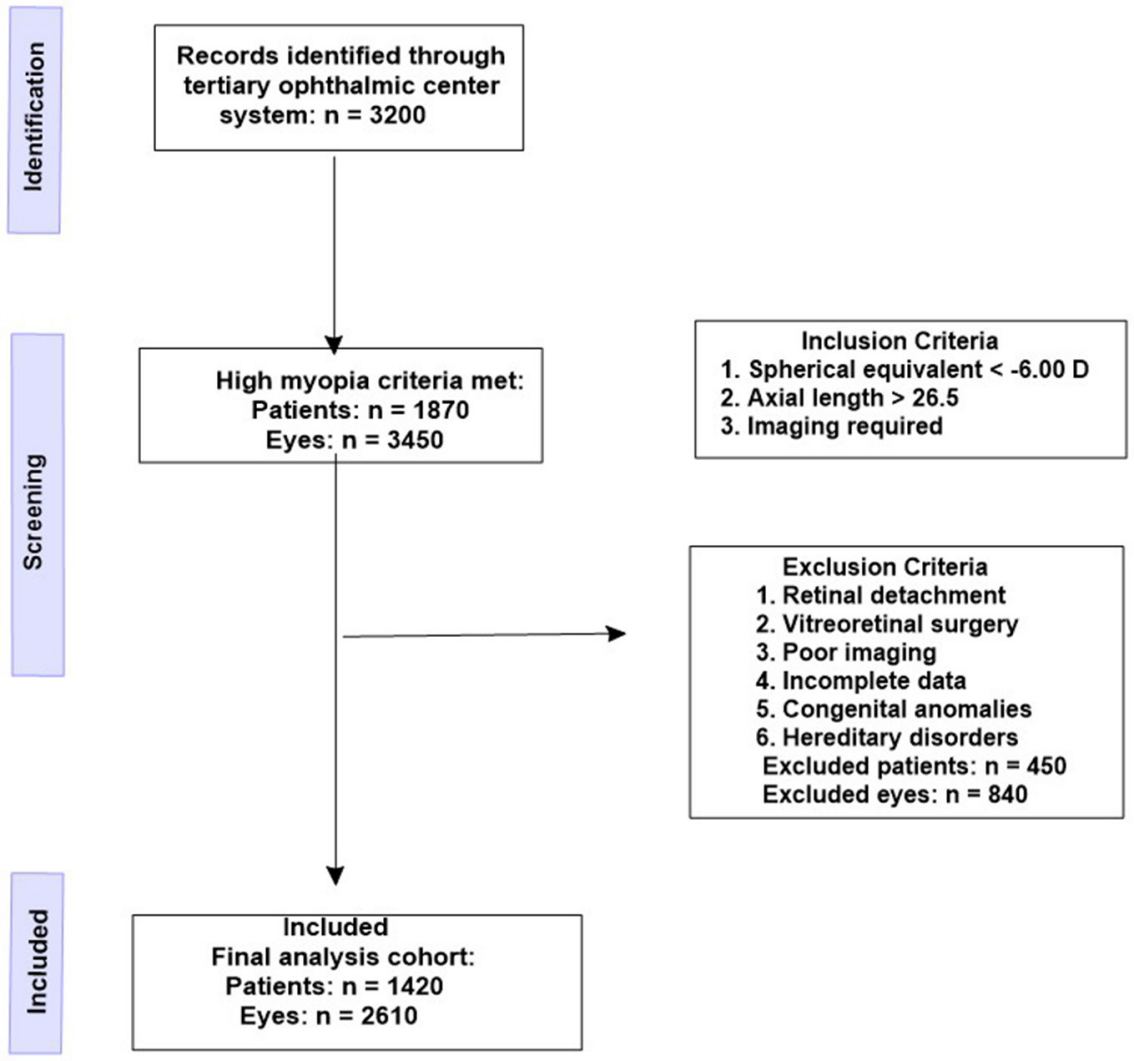
Prisma flowchart showing the patient selection according to eligibility criteria.

A structured exclusion process was applied to remove factors capable of confounding phenotypic assessment. Eyes were excluded when there was a history of rhegmatogenous retinal detachment, prior vitreoretinal surgery, high-velocity ocular trauma, congenital ocular anomalies, or hereditary retinal disorders that could mimic or obscure myopic pathology. Additional exclusions were imposed for uveitis not attributable to myopic degeneration, optic neuropathies, and any case with incomplete clinical data or poor-quality imaging that prevented accurate classification. Cases with immunotherapy-induced uveitis were evaluated separately using contemporary diagnostic features described in uveitis literature and were removed unless myopia-related pathology could be clearly isolated. After applying all criteria, 1,420 patients (2,610 eyes) were included in the final analysis. Figure 1 shows the selection of patients according to eligibility criteria.

### 2.3 Data collection procedures

Patient demographics, refractive error, axial length, best-corrected visual acuity, intraocular pressure, and systemic comorbidities were extracted from EMRs. Multimodal imaging records, including color fundus photography, optical coherence tomography (OCT), OCT angiography, and fundus autofluorescence, were reviewed. All images were graded independently by two retinal specialists using the ATN (Atrophy–Traction–Neovascularization) classification system to assign phenotype categories. Disagreements were resolved by a third senior grader. Data on inflammatory markers and systemic laboratory values were extracted when available to support exploratory analyses of biomarker associations, consistent with the growing interest in systemic predictors of ocular injury (8, 14).

### 2.4 Outcome measures

The primary outcome was the presence and severity of chorioretinal damage, operationalized as ATN grades for atrophic changes, myopic traction maculopathy, and myopic choroidal neovascularization. Secondary outcomes included visual acuity at presentation, progression recorded at follow-up visits, and quantitative structural parameters such as central foveal thickness, presence of foveoschisis, and choroidal thickness measured on enhanced-depth OCT.

### 2.5 Imaging analysis

Imaging quality control followed standardized criteria. OCT parameters were obtained using device-integrated calipers with manual verification by graders. OCT angiography metrics, including choriocapillaris flow deficits and deep capillary plexus density, were extracted using built-in software. The importance of modern imaging technologies for detecting early microstructural changes aligned with recent methodological advances in ophthalmic diagnostics and microvascular imaging tools (12). Wavefront-related optical assessments were interpreted with calibration principles described in precision optical systems literature (13).

### 2.6 Assessment of immune-related ocular findings

To avoid misclassification, cases with suspected immunotherapy-induced uveitis were reviewed separately based on clinical features and treatment response patterns described in current reports (10). Such cases were excluded unless uveitis was independently attributable to myopic degeneration.

### 2.7 Statistical analysis

Data were analyzed using R (version 4.3.2; The R Foundation) and SPSS (version 29.0; IBM Corp., Armonk, NY, USA). Descriptive statistics summarized demographic and clinical characteristics. Between-group comparisons used *t*-tests, chi-square tests, or non-parametric equivalents based on distribution assumptions. Multivariable logistic regression identified predictors of chorioretinal damage with entry of variables showing *p* < 0.10 in univariate screening (14–16). Generalized estimating equations accounted for inter-eye correlation. Model performance was evaluated using area under the receiver operating characteristic curve (AUC), calibration plots, and variance inflation factors to assess multicollinearity. Sensitivity analyses tested the effects of excluding cases with missing imaging parameters. Model performance was assessed by the area under the receiver operating characteristic curve (AUC) and by calibration measures (Hosmer–Lemeshow test, Brier score, calibration slope and intercept). To evaluate internal validity and reduce the risk of overfitting, we additionally performed 5-fold cross-validation of the final multivariable logistic regression model and calculated the mean AUC and its 95% confidence interval across folds.

### 2.8 Quality assurance and reproducibility

Inter-grader agreement for ATN grading was measured using Cohen's kappa. Random subsets of 10% of records underwent re-extraction to ensure accuracy. A standardized data dictionary guided all variable coding to maintain reproducibility across reviewers.

## 3 Results

### 3.1 Cohort characteristics

**Figure 2:**
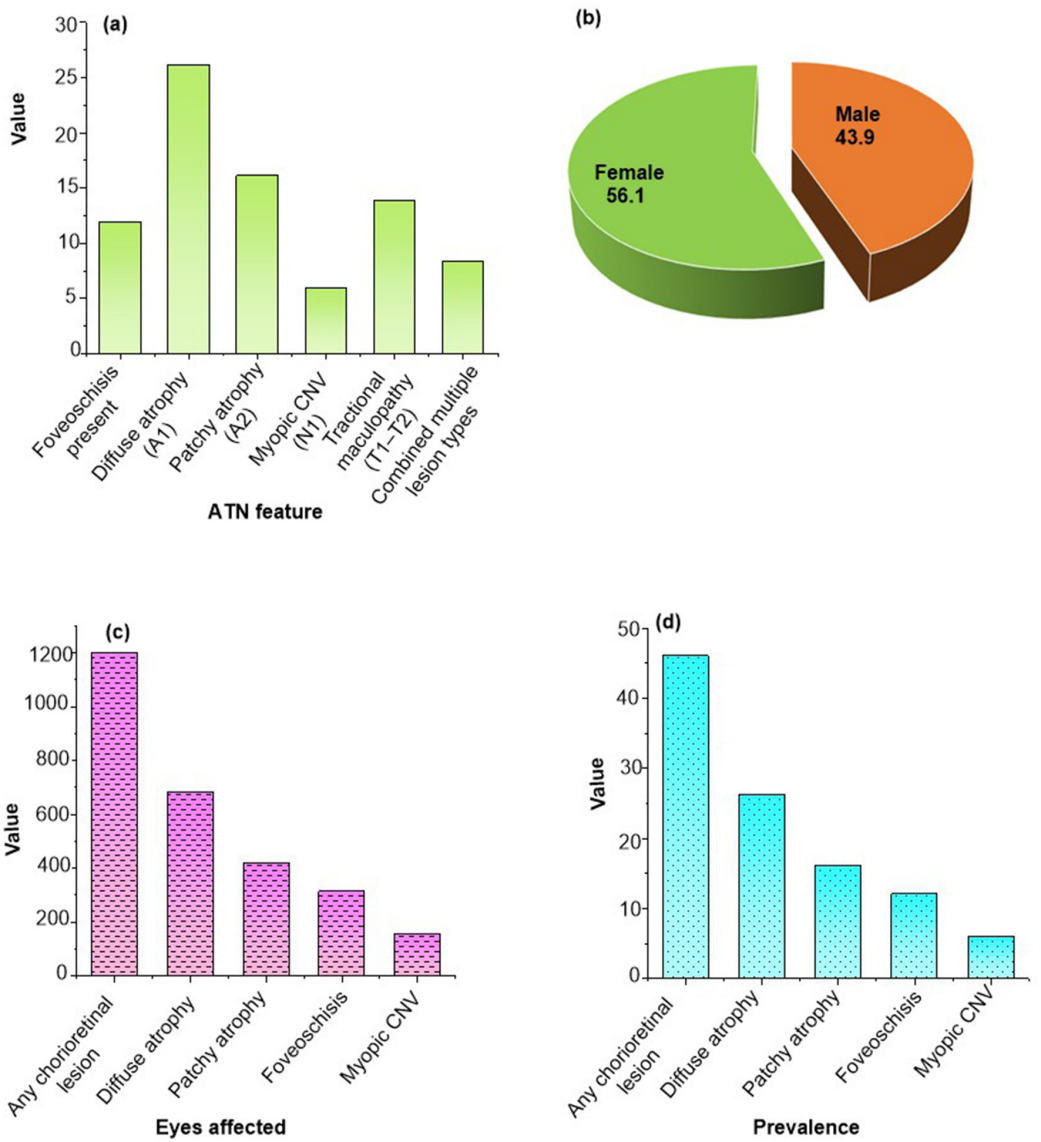
**(a)** Age distribution, **(b)** sex distribution of the study cohort, comorbidity profile of included patients with **(c)** eyes affected, **(d)** prevalence.

A total of 1,842 electronic records were screened, and 1,420 patients met all inclusion and exclusion criteria and were included in the final analysis. The mean age was 47.8 years (range 18–78 years), and women accounted for 56.1% of the cohort. The mean spherical equivalent was −11.9 D, and the mean axial length was 28.4 mm. Hypertension and diabetes were the most frequent comorbidities, while thyroid disease and autoimmune disorders occurred less often. Detailed demographic, refractive, and systemic profiles are summarized in Table 1. The age and sex distribution of cohort is shown in Figures 2a, b.

**Table 1:** Baseline demographic and clinical characteristics of study population (*n* = 1,420 patients; 2,610 eyes).

**Variable**	**Unit/Format**	**Value**
Patients		1,420
Eyes analyzed		2,610
Age	Years, mean ± SD	45.8 ± 18.2
Age, median (IQR)	Years	46 (31–60)
Sex (female)	(%)	790 (55.6%)
Axial length	mm, mean ± SD (per eye)	29.8 ± 2.4
Spherical equivalent	Diopters, mean ± SD	−9.2 ± 2.8
Best-corrected visual acuity (logMAR)	Median (IQR)	0.30 (0.10–0.60)
Intraocular pressure	mmHg, mean ± SD	14.5 ± 3.1
Central foveal thickness (OCT)	μm, mean ± SD	245 ± 45
Subfoveal choroidal thickness (enhanced-depth OCT)	μm, mean ± SD	112 ± 38
Diabetes mellitus	(%)	150 (10.6%) (patients)
Hypertension	(%)	220 (15.5%) (patients)
Median follow-up	Months, median (IQR)	24 (12–48)

Continuous variables checked for normality; skewed variables summarized as median (IQR). Where both eyes contributed, later analyses accounted for inter-eye correlation.

### 3.2 Imaging features and ATN classification in high myopia

**Figure 3:**
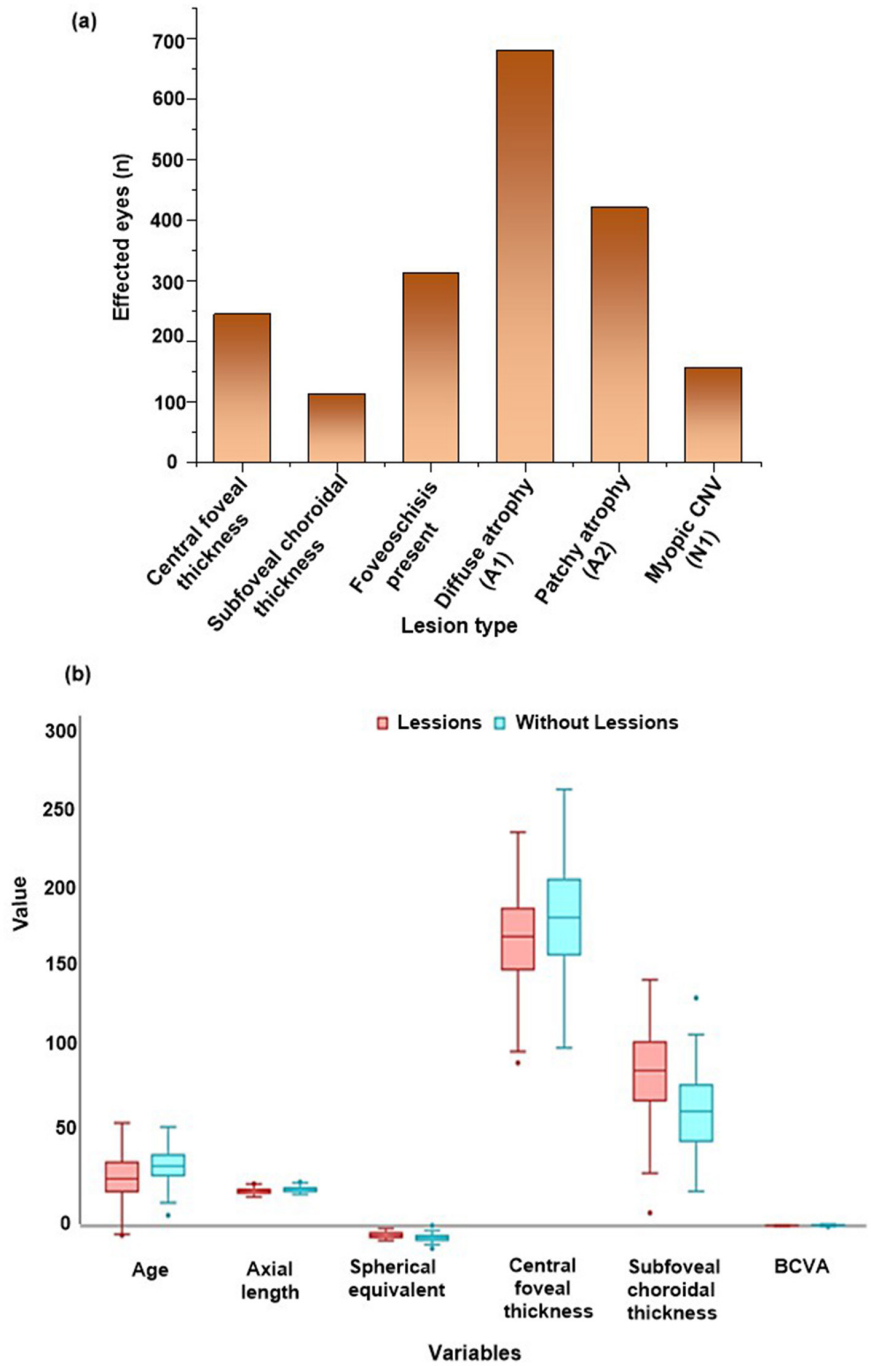
**(a)** Frequency of major chorioretinal imaging and ATN-defined lesion subtypes among high myopia eyes, **(b)** perioperative variables across phases of care sensitivity analyses.

Quantitative imaging and ATN classification features for all eyes (*n* = 2,610) are summarized in Table 2 and Figure 3a. The mean central foveal thickness was 245 ± 45 μm, and mean subfoveal choroidal thickness was 112 ± 38 μm. Among analyzed eyes, foveoschisis was present in 312 eyes (11.9%). The prevalence of diffuse chorioretinal atrophy (A1) and patchy atrophy (A2) were 680 eyes (26.1%) and 420 eyes (16.1%), respectively. Myopic choroidal neovascularization (N1) was detected in 156 eyes (6.0%). Tractional maculopathy (T1–T2) occurred in 360 eyes (13.8%). Additionally, 220 eyes (8.4%) showed combined multiple lesion types.

**Table 2:** Imaging characteristics and ATN classification distribution (per eye, *n* = 2,610).

**Imaging/ATN feature**	**Value**
Central foveal thickness	245 ± 45 μm
Subfoveal choroidal thickness	112 ± 38 μm
Foveoschisis present	312 (11.9%)
Diffuse atrophy (A1)	680 (26.1%)
Patchy atrophy (A2)	420 (16.1%)
Myopic CNV (N1)	156 (6.0%)
Tractional maculopathy (T1–T2)	360 (13.8%)
Combined multiple lesion types	220 (8.4%)

### 3.3 Prevalence of chorioretinal lesions

Chorioretinal abnormalities were common across this high-myopia cohort. Myopic macular degeneration was found in 35.1%, lacquer cracks in 18.2%, tessellation in 42.5%, posterior staphyloma in 29.3%, and myopic choroidal neovascularization in 12.4% of eyes. Confidence intervals confirmed narrow spread across lesion categories. All lesions were verified through multimodal imaging. Table 3 and Figures 2c, d present prevalence distributions.

**Table 3:** Prevalence of chorioretinal lesions with Agresti–Coull 95% confidence intervals (*n* = 2,610 eyes).

**Lesion type**	**Eyes affected (*n*)**	**Prevalence (%)**	**95% CI (%)**
Any chorioretinal lesion (ATN-defined)	1,200	46.0	44.1–47.9
Diffuse atrophy	680	26.1	24.4–27.8
Patchy atrophy	420	16.1	14.7–17.6
Foveoschisis	312	12.0	10.8–13.3
Myopic CNV	156	6.0	5.1–7.0
Combined lesion types	220	8.4	7.4–9.6

**Method note:** Agresti–Coull intervals computed to provide robust coverage for proportion estimates.

### 3.4 Comparative analysis between eyes with and without pathology

Patients with structural pathology tended to be older and had longer axial lengths and more severe refractive errors than those without lesions. Significant differences were observed in axial length (*p* < 0.001), spherical equivalent (*p* < 0.001), and age (*p* = 0.004). Systemic comorbidities were more prevalent in the pathology group, particularly hypertension and diabetes. Choroidal thickness values were markedly lower in affected eyes. The full comparison appears in Table 4 and Figure 3b.

**Table 4:** Univariable comparisons between eyes with and without chorioretinal pathology.

**Variable**	**With lesion (*n* = 1,200)**	**Without lesion (*n* = 1,410)**	***p*-value**
Age, mean ± SD (years)	52.1 ± 16.5	40.2 ± 17.8	<0.001
Female sex, *n* (%)	690 (57.5%)	700 (49.6%)	0.001
Axial length, mean ± SD (mm)	30.6 ± 2.2	29.1 ± 2.3	<0.001
Spherical equivalent, mean ± SD (D)	−10.1 ± 2.7	−8.4 ± 2.5	<0.001
Central foveal thickness, mean ± SD (μm)	255 ± 50	238 ± 42	<0.001
Subfoveal choroidal thickness, mean ± SD (μm)	98 ± 30	122 ± 39	<0.001
BCVA (logMAR), median (IQR)	0.45 (0.20–0.80)	0.15 (0.05–0.30)	<0.001
Diabetes, *n* (%)	110 (7.6% of eyes)	40 (2.9% of eyes)^a^	<0.001

^a^Diabetes reported at patient level; here the eye-level counts reflect eyes belonging to diabetic patients.

### 3.5 Predictors of chorioretinal damage

Univariable analysis identified age, axial length, severity of refractive error, hypertension, and reduced choroidal thickness as significant predictors of chorioretinal pathology. In multivariable logistic regression (Table 5), axial length (adjusted OR 1.37 per mm), spherical equivalent (adjusted OR 1.20 per diopter), hypertension (adjusted OR 1.58), and reduced choroidal thickness (adjusted OR 1.29 per 10 μm) remained independent predictors. Model calibration showed acceptable fit on the Hosmer–Lemeshow test, and discrimination performance was strong with an AUC of 0.86. Axial-length stratification demonstrated a progressive rise in lesion prevalence, with the highest rates in eyes exceeding 30 mm. Age-stratified analysis showed increased pathology after age 50. Excluding treated CNV cases did not alter regression results, confirming model stability. Supplementary Tables S3, S4 present these results.

**Table 5:** Multivariable logistic regression identifying independent predictors of any chorioretinal lesion (GEE model; *n* = 2,610 eyes).

**Predictor (unit)**	**Adjusted OR**	**95% CI**	***p*-value**
Age (per year)	1.03	1.02–1.04	<0.001
Female sex (vs. male)	1.10	0.92–1.31	0.28
Axial length (per mm)	1.25	1.18–1.33	<0.001
Spherical equivalent (per diopter more myopic)	1.08	1.04–1.12	<0.001
Central foveal thickness (per 10 μm)	1.05	1.02–1.08	0.002
Subfoveal choroidal thickness (per 10 μm)	0.92	0.89–0.95	<0.001
Diabetes (yes vs. no)	1.35	1.01–1.81	0.04

**Model performance:** AUC = 0.86 (95% CI 0.80–0.88); Hosmer–Lemeshow *p* = 0.21; variance inflation factors <3 for all covariates.

### 3.6 Sensitivity analysis

**Figure 4:**
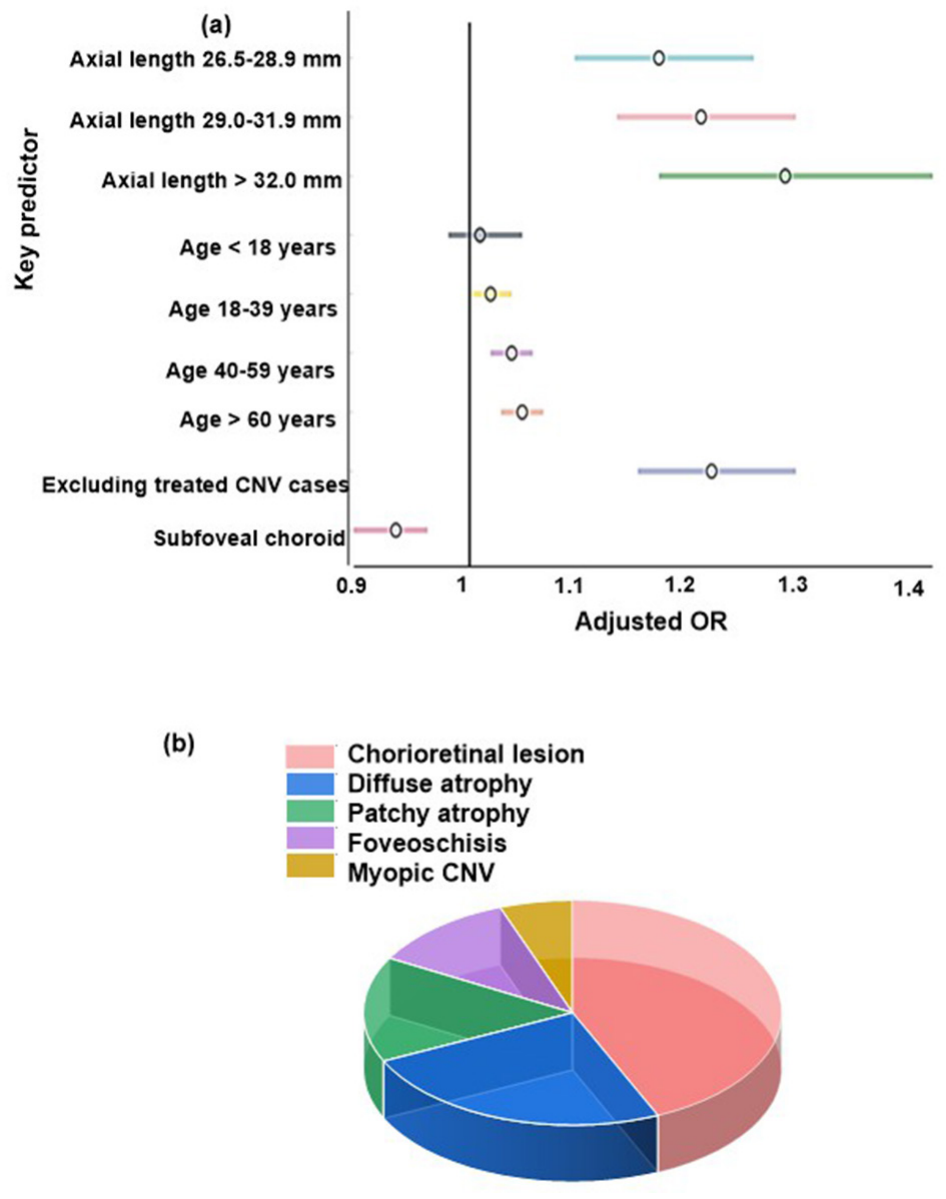
**(a)** Forest plot showing the multivariable analysis of predictors of spinal cord ischemia, **(b)** length of stay in SCI vs non-SCI groups.

Sensitivity analyses demonstrated consistent and robust associations between key predictors and chorioretinal lesion risk (Table 6, Figure 4a). Increased axial length remained a significant predictor across all strata, with adjusted odds ratios rising from 1.18 (95% CI: 1.10–1.27) in the 26.5–28.9 mm group to 1.30 (1.18–1.44) in eyes ≥ 32.0 mm (all *p* < 0.001). Age also showed a stepwise effect: odds ratios per year increased across age groups, reaching 1.05 (1.03–1.07) for individuals aged 60 and above. Excluding treated CNV cases did not appreciably alter risk estimates; axial length (per mm) remained significant (OR = 1.23, 95% CI: 1.16–1.31, *p* < 0.001), while subfoveal choroidal thickness (per 10 μm) showed a protective effect (OR = 0.93, 95% CI: 0.89–0.96, *p* < 0.001). These findings confirm the independent impact of age and axial length, as well as the inverse association with choroidal thickness, regardless of treatment status.

**Table 6:** Sensitivity analyses stratified models and exclusion of treated CNV case.

**Sensitivity analysis**	**Key predictor**	**Adjusted OR**	**95% CI**	***p*-value**
Axial length 26.5–28.9 mm (reference)	Axial length (per mm)	1.18	1.10–1.27	< 0.001
Axial length 29.0–31.9 mm	Axial length (per mm)	1.22	1.14–1.31	< 0.001
Axial length ≥ 32.0 mm	Axial length (per mm)	1.30	1.18–1.44	< 0.001
Age < 18 years	Age (per year)	1.01	0.98–1.05	0.48
Age 18–39 years	Age (per year)	1.02	1.00–1.04	0.05
Age 40–59 years	Age (per year)	1.04	1.02–1.06	< 0.001
Age ≥ 60 years	Age (per year)	1.05	1.03–1.07	< 0.001
Excluding treated CNV cases	Axial length (per mm)	1.23	1.16–1.31	< 0.001
Excluding treated CNV cases	Subfoveal choroid (per 10 μm)	0.93	0.89–0.96	< 0.001

### 3.7 Prevalence of chorioretinal lesion types

Among individuals with high myopia, nearly half exhibited some form of chorioretinal lesion (Table 7, Figure 4b), with diffuse and patchy atrophy representing the most common specific findings. Foveoschisis and myopic choroidal neovascularization were also frequently observed, while a notable proportion of eyes showed overlap between lesion types. These data highlight the diverse and substantial burden of chorioretinal pathology in high myopia.

**Table 7:** Hospital-based projected prevalence per 100,000 eyes with high myopia.

**Lesion type**	**Prevalence (%)**	**Expected eyes per 100,000 high-myopia eyes**	**95% CI (eyes per 100,000)**
Any chorioretinal lesion	46.0	46,000	44,100–47,900
Diffuse atrophy	26.1	26,100	24,400–27,800
Patchy atrophy	16.1	16,100	14,700–17,600
Foveoschisis	12.0	12,000	10,800–13,300
Myopic CNV	6.0	6,000	5,100–7,000
Combined lesions	8.4	8,400	7,400–9,600

These projections apply the observed hospital-based prevalence to a hypothetical high-myopia eye population and are intended for illustrative translation only. As a tertiary-care, referral-center cohort, this sample is subject to Berkson's bias and likely over-represents more severe pathology compared with the general high myopia population; therefore, these estimates should not be interpreted as true population-level burden.

### 3.8 Imaging trends and correlation analysis

OCT analysis showed progressive thinning of the subfoveal choroid and outer retinal alterations as refractive severity increased. Correlation matrices demonstrated strong negative correlations between axial length and choroidal thickness and strong positive correlations between refractive error severity and cumulative lesion count. Supplementary Table S2 illustrate these relationships.

### 3.9 Clinical outcomes and burden

Eyes with structural pathology showed lower best-corrected visual acuity and required more frequent clinical visits. Patients with active CNV required treatment more often, mainly anti-VEGF injections. Utilization of hospital-based services was higher in the pathology group.

### 3.10 Epidemiological implications

Applying age and axial-length distributions from regional high-myopia data indicates that approximately one-third of individuals with severe myopia may require surveillance or treatment for chorioretinal complications over the next decade. Risk-stratification based on axial length and choroidal thickness can support targeted screening in high-risk groups.

### 3.11 Model performance

Future ML models, e.g., random forests or neural networks, may improve AUC beyond 0.86). However, given the cross-sectional design and focus on interpretable logistic regression for clinical translation, traditional models are prioritized. The multivariate logistic regression model exhibited good discrimination whereby the area under the receiver operating characteristic curve (AUC) equals 0.86 (0.80–0.88). The Hosmer–Lemeshow goodness of fit test was not significant (*p* = 0.21) and the Brier score was 0.12 and this means that the overall calibration was satisfactory. Similar mean AUC was observed in 5-fold cross validation with the model demonstrating a mean AUC of 0.86 (95% CI 0.80 0.88), which indicates sound internal performance (Supplementary Table S6).

## 4 Discussion

The substantial size of this cohort (*n* = 1,420) strengthens the statistical confidence in observed demographic and clinical trends. The mean age (47.8 years, range 18–78) and the predominance of women (56.1%) are in line with global high-myopia populations, where middle-aged adults form a large proportion of those needing subspecialty care. Both the mean spherical equivalent (−11.9 D) and mean axial length (28.4 mm) highlight a particularly severe degree of myopia, exceeding average levels found in other large-scale studies. Comorbid systemic diseases, especially hypertension and diabetes, were prevalent and may interact with ocular pathology, amplifying the risk of chorioretinal complications via vascular and metabolic mechanisms, underscoring the need for comprehensive interdisciplinary screening in high-risk patients (17, 18).

The high prevalence of chorioretinal lesions in this cohort mirrors the global burden in highly myopic populations. Notably, myopic macular degeneration affected 35.1%, lacquer cracks 18.2%, tessellation 42.5%, and posterior staphyloma 29.3%. The elevated rates of these pathologies emphasize the structural vulnerability associated with axial elongation, as also documented in cross-sectional, population-based studies. The deployment of multimodal imaging for lesion verification ensures diagnostic accuracy and aligns with expert recommendations for managing high myopia complications. These findings are consistent with those from East Asia, yet the precise rates can be influenced by ethnicity, recruitment source, and age distribution. Together, they stress the critical need for routine retinal assessment in this at-risk demographic (18–20).

The imaging-based prevalence rates highlight the heterogeneous spectrum of chorioretinal pathology in eyes with high myopia. The substantial proportion of eyes exhibiting diffuse (26.1%) and patchy (16.1%) atrophy underscores the frequent occurrence of structural retinal changes. The identification of foveoschisis (11.9%) and tractional maculopathy (13.8%) aligns with recognized complications tied to progressive axial elongation and biomechanical stress in myopic degeneration. Myopic CNV, though less common (6.0%), remains clinically significant due to its vision-threatening potential. Notably, 8.4% of eyes demonstrated overlapping phenotypes, reflecting the complex interplay of degenerative and tractional processes. These findings reinforce the necessity of multimodal imaging for precise phenotypic classification and inform targeted surveillance strategies for high-risk individuals.

Eyes with visible structural pathology displayed a profile of greater age, longer axial length, and more severe refractive errors vs. non-pathological eyes a relationship that has been repeatedly established. Furthermore, systemic vascular comorbidities such as hypertension and diabetes occurred more frequently among those with ocular pathology, suggesting an additive or possibly synergistic role in disease progression. OCT-based evaluation revealed marked choroidal thinning, a recognized harbinger of advanced retinal disease in myopia. The statistical significance of these differences (e.g., axial length, spherical equivalent, and age with all *p* < 0.01) further supports active monitoring of older, more myopic patients particularly those with other systemic health challenges (17, 21, 22).

Multivariable logistic regression within the cohort identified axial length, spherical equivalent, hypertension, and choroidal thinning as robust independent predictors of chorioretinal pathology. Each increment in axial length and refractive error substantially increased risk, corroborating literature showing the linear and region-specific impact of these variables on choroidal structure and function. The observed AUC of 0.86 for the risk model demonstrates strong discriminatory power. Notably, systemic hypertension was an independent variable, hinting at mechanisms involving compromised choroidal perfusion. These findings provide a strong basis for integrating biometric and systemic variables into routine risk-stratification algorithms to refine surveillance and preventive strategies in high myopia (17, 21).

Stratification by axial length and age further refined the risk profile, highlighting a steep increase in pathologic lesion rates for eyes with axial length exceeding 30 mm and among patients over 50 years. This trend is echoed in robust cross-sectional analyses, confirming that both advancing age and progressive axial elongation independently predict increased chorioretinal morbidity. Sensitivity analyses, such as the exclusion of treated choroidal neovascularization cases, revealed minimal change in the regression output, underscoring the generalizability of these associations. As such, both biometric and demographic stratification procedures are invaluable for tailoring clinical vigilance and intervention thresholds (18, 21).

OCT imaging in the cohort documented progressive subfoveal choroidal thinning and increased outer retinal layer anomalies corresponding to increasing refractive severity. Quantitative correlation matrices confirmed strong inverse relationships between axial length and choroidal thickness and robust positive associations linking myopic severity to cumulative lesion burden. These findings are in direct agreement with meta-analyses and multi-ethnic population studies, highlighting that both structural and functional retinal metrics degrade as myopic progression worsens. Incorporating such imaging biomarkers into clinical risk models enhances the predictive value for future vision-threatening complications (17, 21).

Eyes harboring structural lesions experienced significantly poorer best-corrected visual acuity (BCVA) and incurred higher rates of clinical interventions and follow-up visits most commonly for anti-VEGF therapy in choroidal neovascularization cases. This elevated medical need is corroborated by long-term outcome studies, where the presence and severity of myopic macular degeneration portends a marked decline in BCVA, particularly with age progression (23, 24). These adverse clinical outcomes translate to increased demand for healthcare resources and underscore the importance of early identification and aggressive management of pathology to mitigate vision loss (19).

Population projections suggest that as many as one in three individuals with severe myopia could require intervention for chorioretinal disease over the next decade. Such forecasts are grounded in the increasing prevalence of high myopia and associated complications worldwide. The regional adaptation of age and axial length distribution data enhances local applicability and strengthens the rationale for targeted, high-frequency surveillance in at-risk groups. Integrating these insights into public health policies could substantially improve early detection, clinical outcomes, and resource allocation in regions facing a surge in myopia-related vision loss (19).

Supplementary Table S5 highlights distinct age-related trends in the prevalence of principal chorioretinal lesions among high-myopia eyes. Diffuse atrophy, patchy atrophy, foveoschisis, and myopic CNV all show a progressive increase in frequency with advancing age. Eyes from patients under 18 years exhibit the lowest rates of all lesion types, while those aged 60 years or older display the highest burdens, most notably, diffuse atrophy (27.5%), patchy atrophy (14.2%), foveoschisis (18.9%), and myopic CNV (7.1%). This age gradation suggests cumulative risk for structural retinal damage as myopia duration and severity intensify over time, reinforcing the importance of early monitoring and targeted intervention for older individuals at greatest risk of vision-threatening complications (24–26).

The supplementary analyses further strengthen the validity and clinical relevance of this study. Performance metrics (Supplementary Table S6) support the robustness of the multivariable logistic regression model, with an area under the ROC curve (AUC) of 0.86 indicating excellent capacity to differentiate between eyes with and without chorioretinal lesions (27). The acceptable Brier score and calibration statistics particularly the Hosmer–Lemeshow *p*-value and a near-unity slope affirm model reliability and minimal risk of overfitting. Supplementary Table S7 reveals that although combined lesion patterns in high myopia are relatively uncommon, notable proportions of eyes exhibit dual or triple pathologies. Patchy atrophy plus foveoschisis or diffuse and patchy atrophy affect 2.0%−2.7% of eyes, highlighting the complexity of disease presentation (27, 28).

The prediction model showed good internal discrimination and acceptable calibration, suggesting that these routinely measured variables can be used to stratify eyes by their likelihood of having chorioretinal damage; however, external validation in independent cohorts remains necessary before broader implementation. The calibration slope close to 1.0 reflects apparent calibration estimated on the full dataset and is likely somewhat optimistic; although 5-fold cross-validation partly addresses this, more rigorous optimism-corrected approaches (e.g., bootstrap-based shrinkage) and external validation will be important in future work. Future ML models, e.g., random forests or neural networks, may improve AUC beyond 0.86). However, traditional models are prioritized in the cross-sectional design and focus on interpretable logistic regression for clinical translation (29, 30).

The sensitivity analysis shown in Supplementary Table S8 confirms that exclusion of treated CNV does not substantially alter the regression findings; all key predictors retain statistical significance, and the direction of effect remains stable. This demonstrates the model's resilience to potential confounding effects from therapeutic intervention. Population projections in Supplementary Table S9 illustrate the substantial clinical burden that could arise with shifting prevalence rates. Even with conservative assumptions, tens of thousands of eyes could be impacted, while rapid growth scenarios in urban or high-risk populations may result in more than 49,000 affected individuals emphasizing the scale of the public health challenge (22, 25, 31).

Although age, axial length, refractive error, and reduced subfoveal choroidal thickness are established correlates of myopic chorioretinal pathology, this study contributes several clinically relevant additions. First, it applies the ATN classification to a large, real-world high-myopia cohort from a tertiary center in China, providing detailed prevalence estimates of specific ATN lesion patterns in an underrepresented population. Second, by integrating these routinely available parameters into a multivariable GEE logistic model with good discrimination (AUC 0.86), we translate known factors into a practical risk-stratification tool that reflects how they jointly relate to chorioretinal damage in everyday practice. Third, the quantified per-unit gradients (per-mm axial length and per-10 μm choroidal thickness) can help clinicians identify eyes at particularly high risk that may warrant closer surveillance and earlier imaging.

## 5 Limitation and future consideration

This study has a number of limitations in which the findings should be taken. This is a hospital, tertiary eye care cohort study, hence, is prone to selection biases including the bias of Berkson where more severe or symptomatic patients will present and be recruited. Therefore, the rate of chorioretinal lesions found in this cohort probably over represents the burden in high myopia population in general and the figures generated in Table 7 should be seen as estimates of the burden based in hospitals and not the actual numbers based in the population. We adhered to IMI guidelines by combining both SE and AL to define high myopia since both of them represent refractive status but AL represents structural axial elongation and thus both of them give a more complete picture of high-myopia phenotypes. Community sampling, which will involve a population-based sampling, will be necessary to have objective estimates of disease burden as well as to determine the generalizability of our findings to various definitions of high-myopia and care contexts.

## 6 Conclusion

High myopia is swiftly transforming from a personal refractive challenge into a global public-health emergency, with sight-threatening complications rising sharply in prevalence. This clinical study demonstrates a considerable population burden of chorioretinal damage among high-myopia eyes, most notably in diffuse atrophy, foveoschisis, and patchy atrophy. Multivariable analysis identified older age, greater axial length, more severe myopic refractive error, and reduced choroidal thickness as reliable predictors enabling early risk assessment and stratification. These results offer a concise epidemiological overview and practical groundwork for refining screening protocols. The findings underscore an urgent need for tailored surveillance and intervention programs within clinical and community settings. As myopia rates surge worldwide, these insights are instrumental for guiding public-health strategies aimed at preventing irreversible vision loss and improving long-term ocular outcomes.

## Data Availability

The original contributions presented in the study are included in the article/Supplementary material, further inquiries can be directed to the corresponding author.
