# Supplementary material for: Clinical spectrum and prevalence associations of chorioretinal damage in high myopia: a retrospective cross-sectional analysis
Source: Front Med (Lausanne). 2026 Feb 17;13:1752116. doi: 10.3389/fmed.2026.1752116 (PMC12953075; doi:10.3389/fmed.2026.1752116)
Supplement: Supplementary file 1 [file Table_1.docx]

**Clinical Spectrum and Prevalence Associations of Chorioretinal Damage in High Myopia: A Retrospective Cross-Sectional Analysis**

**Table S1:** **Stepwise selection of eyes and patients from initial screening to final inclusion**

| **Screening Stage** | **Patients (n)** | **Eyes (n)** | **Notes** |
| --- | --- | --- | --- |
| Total myopia records screened (2015–2024) | 3,200 | 5,980 | All refractive error categories |
| High myopia preliminary pool | 1,870 | 3,450 | Based on SE ≤ −6.00 D or AL ≥ 26.5 mm |
| Exclusions due to incomplete imaging | 210 | 380 | Poor OCT/Fundus quality |
| Exclusions: confounding ocular disease | 140 | 260 | RD history, trauma, hereditary disorders |
| Exclusions: uveitis and unclear etiology | 100 | 200 | Following immunotherapy-induced uveitis criteria |
| Final cohort included | 1,420 | 2,610 | Used for all analyses |

For the present analysis, only baseline (first‑visit) data from the final cohort (1,420 patients; 2,610 eyes) were used in a retrospective cross‑sectional design; follow‑up data, although available for some patients, were not analyzed.

**Table S2: Technical specifications of imaging systems used to classify chorioretinal pathology**

| **Imaging Modality** | **Parameter** | **Device/Model** | **Value/Setting** | **Notes** |
| --- | --- | --- | --- | --- |
| OCT | Axial resolution | Spectral-domain OCT | 5–7 μm | Standard protocol |
| OCT | Scan length | 6 mm horizontal/vertical | — |  |
| OCT | Averaging | 20–30 frames | — |  |
| Fundus Photography | Resolution | 45° field | High-res |  |
| OCTA (if available) | Slab segmentation | ILM–RPE | Device default | CNV confirmation |
| Ultra-widefield imaging | Field | 200° | — | Peripheral changes |

**Table S3: ATN Grading Concordance between Two Masked Graders**

| **Parameter** | **Grader A (n)** | **Grader B (n)** | **Agreement (%)** | **Cohen’s κ** |
| --- | --- | --- | --- | --- |
| A-grade (atrophy) | 620 | 610 | 93.1 | 0.89 |
| T-grade (traction) | 310 | 295 | 92.4 | 0.87 |
| N-grade (neovascular) | 98 | 95 | 96.2 | 0.92 |
| Final ATN composite grade | — | — | 91.7 | 0.88 |

**Table S4:** **Demographic and imaging variation across axial length strata**

| **Axial Length Group** | **Eyes (n)** | **Age (years)** | **SE (D)** | **Choroidal Thickness (μm)** | **Lesion Prevalence (%)** |
| --- | --- | --- | --- | --- | --- |
| < 29 mm | 1,050 | 39.2 ± 15.8 | −7.1 ± 2.1 | 92 ± 24 | 23.5 |
| 29–31.9 mm | 980 | 45.3 ± 16.1 | −9.4 ± 2.5 | 70 ± 26 | 41.8 |
| ≥ 32 mm | 580 | 51.6 ± 14.2 | −11.8 ± 3.1 | 46 ± 21 | 68.1 |

**Table S5:** **Variation in lesion type and prevalence across age groups**

| **Age Group** | **Eyes (n)** | **Diffuse Atrophy (%)** | **Patchy Atrophy (%)** | **Foveoschisis (%)** | **Myopic CNV (%)** |
| --- | --- | --- | --- | --- | --- |
| < 18 years | 210 | 4.7 | 1.4 | 6.6 | 0.0 |
| 18–39 years | 960 | 11.3 | 5.2 | 10.1 | 1.1 |
| 40–59 years | 930 | 18.6 | 9.1 | 13.5 | 4.0 |
| ≥ 60 years | 510 | 27.5 | 14.2 | 18.9 | 7.1 |

**Table S6:** Apparent (training‑set) and cross‑validated performance metrics for the final logistic regression model

| **Test** | **Metric** | **Value** | **Interpretation** |
| --- | --- | --- | --- |
| ROC AUC | AUC | 0.86 | Strong discrimination |
| Brier score | Score | 0.12 | Acceptable accuracy |
| Hosmer–Lemeshow test | p-value | 0.41 | Calibration not violated |
| Calibration slope | Slope | 0.98 | Minimal overfitting |
| Calibration intercept | Intercept | 0.04 | Good alignment |

Calibration slope and intercept reported in the main text represent apparent calibration on the full dataset; cross‑validated performance estimates are also provided to illustrate internal variability.

**Table S7: Co-occurrence of chorioretinal lesions in high-myopia eyes**

| **Lesion Combination** | **Eyes (n)** | **Percentage (%)** |
| --- | --- | --- |
| Diffuse + Patchy atrophy | 62 | 2.4 |
| Patchy atrophy + Foveoschisis | 71 | 2.7 |
| Diffuse atrophy + Foveoschisis | 52 | 2.0 |
| Any atrophy + CNV | 35 | 1.3 |
| Triple combination (A + T + N features) | 15 | 0.6 |

**Table S8: Effect of removing treated CNV cases on odds ratios for main predictors**

| **Predictor** | **Adjusted OR** | **95% CI** | **p-value** |
| --- | --- | --- | --- |
| Age (per year) | 1.02 | 1.01–1.03 | <0.001 |
| Axial length (per mm) | 1.55 | 1.38–1.73 | <0.001 |
| SE (D) | 1.07 | 1.04–1.11 | <0.001 |
| Choroidal thickness | 0.98 | 0.97–0.99 | <0.001 |

**Table S9: Projected Population Burden under Different Prevalence Scenarios**

| **Scenario** | **Prevalence (%)** | **Projected Eyes Affected** | **Notes** |
| --- | --- | --- | --- |
| Lower-bound (15%) | 15 | 12,300 | Conservative estimate |
| Observed (approx 40%) | 40 | 32,900 | Based on current study |
| High-prevalence region (50%) | 50 | 41,200 | Urbanized settings |
| Rapid-growth scenario (60%) | 60 | 49,400 | High-risk demographics |
